# Social Stigma of Patients Suffering from COVID-19: Challenges for Health Care System

**DOI:** 10.3390/healthcare10020292

**Published:** 2022-02-02

**Authors:** Magdalena Rewerska-Juśko, Konrad Rejdak

**Affiliations:** Chair and Department of Neurology, Medical University of Lublin, Jaczewskiego 8, 20-954 Lublin, Poland; konrad.rejdak@umlub.pl

**Keywords:** COVID-19, SARS-CoV-2, stereotypes, social stigma, health care system, comorbidities

## Abstract

The meaning of the term social stigma has changed over the years. The history of this concept dates back to ancient times. Currently, social stigma is defined as the attitude of discrimination, disapproval, or negative perception of a given group due to the properties and features it represents. Stigmatization concerns the physical and mental spheres of an individual. The burden of stigma affects many people. Moreover, it is present in medicine, affects people with COVID-19 and presents a challenge for the health care system. Social stigma of individuals with COVID-19 is a worldwide problem and can be compounded by including race, profession, social status, religious identity, and vaccination status. Stigmatization may lead to negative consequences, including discrimination and social rejection of stigmatized individuals. In addition, it affects the close relatives of stigmatized individuals. The main goal of this review paper is to present the problem of stigma among patients suffering from COVID-19 and to list major challenges for the health care system in solving this problem. We undertook a review of literature published in PubMed systems, Scopus and Google Scholar. The results indicate that the stigmatization bears many negative consequences including limited access to health care, potential impact on health status of patients and worse outcomes. Early identification of the problem may help to implement appropriate strategies to combat the stigma.

## 1. Introduction

The meaning of the term “stigmatization” has evolved over the years. The origin of the term dates back to ancient times. The Greek word “stigma” previously referred to marks that were placed on the bodies of individuals considered by a given society to be inferior such as slaves or criminals [1,2]. Currently, “stigmatization” regards the physical and psychological realm of a person. We define it as disapproval, social disgrace and negative perception of a particular group within society due to the traits, value system and other attributes that characterize this group [1,2,3].

The modern concept of “stigma” was introduced by Erving Goffman, who, in 1963 published a book with a title „Stigma: notes on the management of spoiled identity” [1,2,3].

E. Goffman separated “stigma” into three separate groups. The first concerned deformities of the body. The second was related to a person’s psychological sphere, including illnesses, addictions or homosexuality. The last one included race, origin, nationality or religion with respect to stigma [1,2]. The concept of stigma has been considered by other authors in different aspects over the years.

According to the concept of stigma phenomenon created by Bruce Link and Joe Phelan, stigma is present when four elements form the phenomenon. These include:

1. Isolating a difference and giving a label to the person affected by that difference;
2. Linking isolated social categories to negative stereotypes;
3. Separating “us” from “them”—negative stereotyping, influences rejection, exclusion and discrimination;
4. Loss of social status—the final stage of stigmatization, such as deprivation of medical care [3,4].

In turn, other authors distinguished in their work three attitudes of the stigma caused by the disease; (1) a person’s reaction to the illness (auto-stigma); (2) the attitude displayed towards the stigmatized person (personal stigma); (3) beliefs about other people’s attitudes toward stigmatized patients (perceived stigma) [5]. 

As E. Goffman highlights, every individual expects recognition and respect, but a stigmatized person will not experience these ethical considerations. The experience of being stigmatized is typically painful and hinders recovery and daily functioning [1,6].

According to the Health Stigma and Discrimination Framework, stigma associated with health conditions can manifest itself in a wide range of experiences: prejudice experienced by stigmatized people in their society (stigmatized stigma); perceptions of how stigmatized people are treated in a given context (perceived stigma); and expectations of being stereotyped, and discriminated against by others in the future, e.g., if the health condition worsens and is known (anticipated stigma) [7,8].

## 2. Methods

In this article we performed a review of the literature with PubMed, Scopus and Google Scholar. The literature search used the keywords (one or in combinations): COVID-19, SARS-CoV-2, stereotypes, social stigma, health care system, comorbidities. All original and review articles had been searched extensively in the database. A total selection of 53 papers written in the English language were included, excluding duplicates and works written in other languages. Overall, the corresponding articles were reviewed and considered accordingly.

## 3. Results

### 3.1. Stigma and COVID-19

Stigmatization is a widespread phenomenon in the current world. In medicine, the concept of stigma is associated with several diseases. There have been numerous papers describing its impact on patients suffering from mental illnesses, [9,10], psoriasis [11], dementia [2] and after a stroke [12].

Presently, humanity faces one of the greatest challenges of the century. The novelcoronavirus is spreading rapidly, to the extent where it has been declared a worldwide pandemic. COVID-19 is a serious infectious disease that has claimed over 150,000 lives and infected millions in the United States to date, especially in the elderly population. Globally, the spread of the COVID-19 pandemic has instigated fear and raised concerns. There has been a sudden change in people’s daily lives. In addition to fear, anxiety, and sadness, a sense of irritability has begun to develop. Amid such an extreme spread of the SARS-CoV-2 virus, one important issue that is even more damaging than all of the above-mentioned negative effects and requires urgent attention is the stigma associated with the pandemic. People are witnessing a dramatic shift from wanting to engage in relationships with one another to practicing stigmatization of individuals, groups and nations, which is perceived as a potential source of infecting others with the virus [13,14]. Stigmatizing behavior is triggered by fear of the unknown and uncertain. There are negative attitudes towards people who might have been infected, infected individuals, and people perceived to be spreading the virus. The stigma of COVID-19 can be understood as a social process that aims to exclude those who are perceived as a potential source of the disease and who may pose a threat to effective social life in a given society [14,15,16]. Consequently, those who are stigmatized experience shame, anxiety, and fear of rejection as they are unable to meet the expectations of society. Prejudice and discriminatory responses to the stigmatized are of additional concern in the context of pandemics such as acute respiratory distress syndrome [14].

### 3.2. COVID-19 and Comorbidities 

The population of elderly people and people suffering from Alzheimer’s disease and other dementia-related diseases appear to be at a greater risk. Age and comorbidities were consistently the most influential factors correlated with poor prognosis, hospitalization and mortality in patients with COVID-19. Elderly people with dementia associated with Alzheimer’s disease (ADRD) are a particularly vulnerable group as they often have comorbidities [17,18,19]. Moreover, individuals with dementia are more likely to experience strokes, atherosclerosis, diabetes, insomnia, urinary incontinence, fractures and pneumonia compared to individuals of the same age without dementia [20], again putting them at greater risk of developing severe COVID-19 symptoms if they become infected with the virus, leading to higher mortality in this patient population [21].Compliance with public health authorities’ recommendations to reduce the transmission and spread of COVID-19 may not be achievable in ADRD patients for a variety of reasons. People with Alzheimer’s disease may have a higher risk of developing COVID-19 [21]. They may be unable to follow public health recommendations for preventing SARS-CoV-2 infection, such as covering their mouth and nose when coughing, hand hygiene, physically distancing from others or staying at home. These individuals may not understand or remember given instructions. If they are depressed, feel worse, or apathetic, they may become reluctant or unable to follow given rules. Some patients with severe Alzheimer’s disease may be agitated, psychotic or refuse social isolation. Moreover, their behavior may put them at risk of further worsening of their dementia-related condition, particularly if they are in a hospital setting and away from family members or friends. Prolonged hospitalization would bear dire consequences for these individuals. Therefore, caring for Alzheimer’s patients, who are often older and have multiple risk factors, poses a serious challenge to public health, carers, health workers and nursing homes if they become infected with SARS-CoV-2 [21,22]. Doctors must be especially vigilant about COVID-19 problems that could directly impact patient care.

### 3.3. Factors Determining the Stigma of People with COVID-19

The stigmatizing attitude, stereotyping and discrimination can be based on many factors conditioning the stigmatization of these people, including: race, profession, social status, religious identity and lack of vaccination.

Creating a stigmatizing attitude in a given society depends on many circumstances. Race emerged as an important factor influencing the perception of individuals with COVID-19. The observations were made on a population living in the easternmost part of India, including eight states known as Northeast India. The people of Northeast India have long been the target of racism from residents of mainland India as they bear typical Mongoloid traits, similar to those of the Chinese. Residents of Northeast India mostly suffered from the brunt of racism, discrimination and were often considered foreigners in their own country. During the COVID-19 pandemic, racism against Northeast Indians intensified and many cases have been reported where Northeast residents were called “crown”, spit on, asked by landlords to leave their homes, beaten, suspended from work or had difficulty accessing health care [23,24,25]. Literature reports racism as an important factor in the excessive spread of diseases in the minority [26] and even in their death [27]. However, the experience of stigmatization by people from the Northeast in India was mainly due to facial similarities and associations with the Chinese, who are also stigmatized by many as the cause of the pandemic.

Another factor influencing the reception of people with COVID-19 is their respective profession. During the COVID-19 pandemic, countries worldwide are attempting to make the best use of their resources and capabilities to contain the spread of the pandemic. Individuals, groups and communities come together and present examples of pro-social behavior. Among them there are doctors, nurses, psychologists, other health care workers, and policemen who risk their lives to serve their people. Healthcare workers, who work hard to save patients’ lives [28,29,30] and police officers, who work day and night away from their relatives are mistreated by society [31,32,33,34,35]. They experience frustration resulting from their hard struggle and social stigma that ceases to end even with their death [29,36]. These people at the forefront of the war against the pandemic, also colloquially known as “coronavirus warriors”, are ostracized by apartment owners, neighbors, family members and taxi drivers. Healthcare workers had to sleep in staff rooms as taxi drivers additionally refused their service [37]. The ongoing stigmatized behavior against health care workers and uniformed officers illustrates a classic stigma through association [1,38]. In this context, the social stigma becomes a function of unfavorable alliances, in which even people who initially were not part of the stigmatized group (health care workers, uniformed) become the target of stigmatization due to contacts with infected patients [39]. The severity of the phenomenon of stigma additionally depends on other factors. Moreover, there has been a social stigma against some marginalized groups, such as the homeless, the poor and migrant workers. Upon returning home after months of being stuck in different parts of the country, migrant workers and their families were mocked and harassed by members of the community [40]. There have been many cases in various Indian states where people had not reported their travel history or symptoms of COVID-19 due to fear of social boycott and discrimination, leading to reduced testing and high mortality [41]. Homeless and poor people were negatively perceived as infected with the disease, accused of ignorance and neglect, and thus responsible for contracting the virus and treated as active carriers of the coronavirus and as passive buyers of the disease [42]. Religious identity is a factor that may influence the perception of people with COVID-19. In March 2020, a religious meeting of members of the Islamic missionary and reformist organization from around the world was held in Delhi. It was later discovered that most of these members were infected with COVID-19and they returned to their respective places all over India. At the time, fear of spreading the virus among the general public was at its peak and the entire Muslim community was branded as spreaders of the virus [43]. Consequently, an increase in hostility, segregation and violence was directed against the entire Muslim community and contributed to strengthening the preexisting gaps that endure between religious groups in society [44].

Another factor that increases stigma is the issue of vaccination. The decision not to receive vaccination bears both health and social risks for individuals and communities. Individuals who reject, flaunt, defy or go against social expectations, may be subject to intense forms of stigma [45].

### 3.4. Consequences of Stigmatization Due to COVID-19

COVID-19 and the associated stigma affect the occurrence of negative consequences not only for sick people but also for their environment.

“The stigma comes from fear. In turn, fear causes silence, which contributes to the occurrence of misunderstanding and ignorance” [46]. Fear causes anxiety among patients and those around them. Stigmatization may help to hide symptoms related to the disease, which delays the time of seeking appropriate help and implementing treatment [46,47]. This behavior greatly improves the spread of infectious pathogens, especially within people with mild symptoms who behave as usual so as not to arouse suspicion. These people avoid seeking appropriate medical attention. Such behavior may aggravate the clinical condition with serious psychological consequences. Prolonged loneliness and isolation may trigger mental disorders such as depression experienced by some people. A study of 3005 elderly people found that being in isolation increased the prevalence of depressive and anxiety disorders [47]. Conversely, patients diagnosed with COVID-19 who were hospitalized or undertook home quarantine measures often suffered from anxiety and depression due to isolation or feelings of guilt towards family members or other close people. There may also be states of social phobia and xenophobia as a consequence of social stigma [47].

As mentioned above, stigmatization accelerates the spread of infectious diseases. Moreover, it can affect the deterioration of patients’ health status and people’s social and health-seeking behaviors. Individuals with COVID-19, in fear of being stigmatized, and marginalized in society, may manifest behaviors that are detrimental to their health, such as avoiding diagnostic examinations and tests. Stress caused by hiding symptoms of the disease may additionally lead to immunological depression and delays timely diagnosis and implementation of treatment [48,49].

In addition, stigmatization affects the families of patients. Lack of knowledge about the disease can lead to incorrect judgments which affect not only the patient but also their family. People in contact with the sick may themselves become victims of stigmatization. This phenomenon is called “stigma by association” and may also affect partners, friends and medical staff [50]. People in the family of stigmatized people may experience shame, fear, anger, helplessness, or lack of support. The stigma has had a profound effect on the mental health of frontline workers, as well as those recovering or surviving the disease. The media reports on the effects of isolation and discrimination on suicide rates in India. Experiencing isolation, stigmatization and social boycott increased the risk of loneliness and self-harm [51].

### 3.5. Specific Antistigma Interventions and Strategies

Research clearly indicates that the stigma and fear associated with COVID-19 makes it difficult to take appropriate remedial steps. Meanwhile, Adiukwu and co-authors present specific strategies to combat COVID-19 related stigma at institutional, community and individual levels that can be adapted to any phase of the pandemic [52].

**Health Institution Level**

1. Identify factors associated with stigma
2. Identify dominant forms of stigma
3. Develop action plan to help address factors associated with stigma and forms of stigma.
  a. Education on stigma
  b. Provision of personal protective equipment by hospital management
  c. Training in the management of COVID-19 for cadre of health staff
  d. Avoiding marking beds or wards with “COVID-19”
  e. Develop a disciplinary panel for health care workers who stigmatize despite training
  f. Conducting systematic training on the management of an infectious disease with
  g. The potential for global spread
  h. Conducting an anti-stigma campaign for all forms of infectious diseases.

**Community Level**

1. Identify factors associated with stigma
2. Identify dominant forms of stigma
3. Develop action plan to help address factors associated with stigma and forms of stigma.
  a. Train the media to ensure ethical journalism
  b. Identify and correct myths and misinformation
  c. Celebrate memorial days and COVID-19 heroes
  d. Use social influencers
  e. Provide accurate and up to date information concerning COVID-19
  f. Infomercials on COVID-19 should not focus on any particular ethnic or racial group and should be produced in partnership with public figures and celebrities
  g. Provide regular broadcasted messages on infectious disease with potential for global spread
  h. Highlight strengths and positive aspects of the country when providing updates on COVID-19
  i. Put policies in place to help citizens recover from the socioeconomic consequences of the pandemic.

**Individual Level**

1. Identify and respond to the needs of the stigmatized population.
  a. Identify factors associated with stigma
  b. Identify forms of stigma.
    ▪ Ensure confidentiality when requested
    ▪ Provide policies on accessing healthcare post pandemic
    ▪ Provide psychosocial support
    ▪ Support victims of stigma
    ▪ Make healthcare readily accessible and widely available.

In a systematic review of the literature on the effectiveness of interventions to reduce stigma, the World Health Organization (WHO) points to activities related to building trust in health care, providing appropriate and proven information, showing empathy to people affected by the disease and creating an environment in which open discussion between people and health professionals will be facilitated. It is important to promote understanding of the disease itself and to use effective, practical preventive measures to ensure the safety of oneself and one’s loved ones. The way in which we communicate when talking about COVID-19 is critical to helping us take effective action to combat the disease and avoid fueling fear and stigma. An environment should be created, where disease and related problems can be discussed and dealt with in an open, honest and effective manner [39].

### 3.6. List of Major Challenges for the Health Care System in the Era of COVID-19

The COVID-19 pandemic situation forces a number of changes in the functioning of the health care system. It exposes the difficulties that health care has faced so far, generates new problems and verifies the level of preparedness for the emergency situation [53]. Below is a list of the most important actions a health care system can undertake to combat stigma posed by COVID-19:

1. Systematic training of health professionals to assist in the assessment and treatment of people affected by stigma.
2. The establishment of a psychological support network for health workers who are at increased risk of COVID-19related stigma.
3. Implementation of medical IT systems for online patient services (allowing video calls, among other things, psychological support for people who have experienced stigma or discrimination).
4. To develop educational material for patients and the general public on how to combat stigma.
5. Providing information from reliable sources to increase knowledge and reduce stigma associated with COVID-19.
6. Improving access to support and health care
7. Maintaining the confidentiality and privacy of those seeking healthcare.
8. Correcting negative language that can cause stigma.
9. Speaking out against negative statements and behaviors.

## 4. Discussion and Conclusions

Stigmatization is a common phenomenon in the modern world. Despite several years of numerous observations, the specifics of this process still remain unclear. The origins can be traced back to mechanisms in the human psyche that have yet to be fully understood. The problem of social stigmatization affects many people. Moreover, it is present in medicine, and it also concerns patients with COVID-19. The degree of social stigmatization observed in connection with the COVID-19 pandemic is caused by many factors, which are presented in this review paper. Its negative effects can be observed on a daily basis. Stigmatization causes anxiety, fear, lowers self-esteem and impairs the quality of life of stigmatized people. Stigma can contribute to masking symptoms of the disease, discouraging people from seeking immediate medical help and engaging in health-seeking behaviors. Preventing the spread of attitudes and behaviors associated with stigma can help reduce the spread of the COVID-19 pandemic since, as mentioned above, stigma can lead to underreporting of symptoms and reduced use of health facilities. While stigma associated with COVID-19 can cause significant challenges for the health care system, we have outlined a number of ways in which these can be addressed. Addressing these challenges through training, education and support, will reduce the impact of COVID-19 related stigma on individuals and the healthcare system.

In summary, we envisage two general challenges related to stigma among the population for the upcoming years. Firstly, we need to prepare for the appearance of new variants of the virus and repeated waves of local infections in an endemic fashion. Thus, patients affected will face isolation, long term quarantine which poses additional distress and stigma [54]. This is currently reflected in countries with new outbreaks of infection and large scale measures to suppress the spread of the virus.

**Figure 1:**
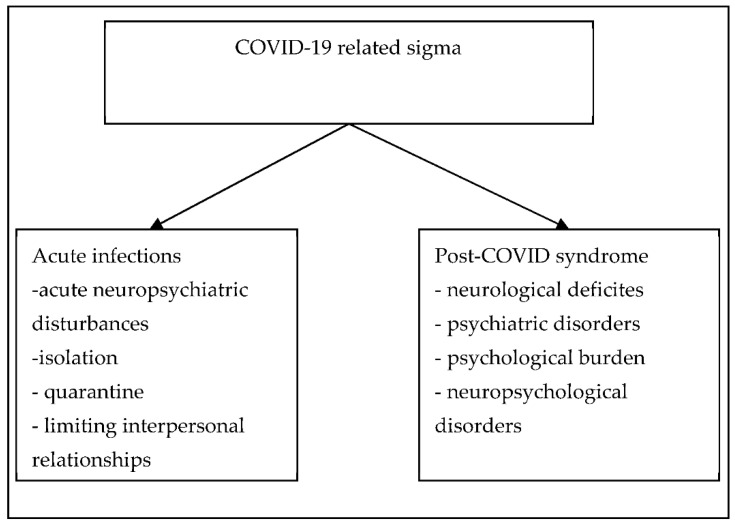
Predicted scenarios for COVID-19 related stigma.

Secondly, we predict that large numbers of patients who recovered from COVID-19 and suffering from long COVID syndrome will also be affected by different health burdens and require appropriate treatment and care [55]. Figure 1 presents predicted scenarios for COVID-19 related stigma.

## Data Availability

Data sharing not applicable.
